# “Mombrain and Sticky DNA”: The Impacts of Neurobiological and Epigenetic Framings of Motherhood on Women's Subjectivities

**DOI:** 10.3389/fsoc.2021.653160

**Published:** 2021-04-13

**Authors:** Ingrid Olivia Norrmén-Smith, Ana Gómez-Carrillo, Suparna Choudhury

**Affiliations:** ^1^Division of Social and Transcultural Psychiatry, Department of Psychiatry, McGill University, Montréal, QC, Canada; ^2^Institute of Community and Family Psychiatry, Lady Davis Institute for Medical Research, Jewish General Hospital, Montréal, QC, Canada

**Keywords:** neuroscience, epigenetics, knowledge translation, media, pregnancy, motherhood, perinatal period, mental health

## Abstract

The fields of epigenetics and neuroscience have come to occupy a significant place in individual and public life in biomedicalized societies. Social scientists have argued that the primacy and popularization of the “neuro” has begun to shape how patients and other lay people experience themselves and their lifeworlds in increasingly neurological and genetic terms. Pregnant women and new mothers have become an important new target for cutting edge neuroscientific and epigenetic research, with the Internet constituting a highly active space for engagement with knowledge translations. In this paper, we analyze the reception by women in North America of translations of nascent epigenetic and neuroscientific research. We conducted three focus groups with pregnant women and new mothers. The study was informed by a prior scoping investigation of online content. Our focus group findings record how engagement with translations of epigenetic and neuroscientific research impact women's perinatal experience, wellbeing, and self-construal. Three themes emerged in our analysis: (1) A kind of brain; (2) The looping effects of biomedical narratives; (3) Imprints of past experience and the management of the future. This data reveals how mothers engage with the neurobiological style-of-thought increasingly characteristic of public health and popular science messaging around pregnancy and motherhood. Through the molecularization of pregnancy and child development, a typical passage of life becomes saturated with “susceptibility,” “risk,” and the imperative to preemptively make “healthy' choices.” This, in turn, redefines and shapes the experience of what it is to be a “good,” “healthy,” or “responsible” mother/to-be.

## Introduction

In this paper, we set out to analyze women's engagement with nascent epigenetic and neuroscientific bodies of research in North America. This is part of our broader interest about the extent to which, and ways in which, new knowledge related to the brain and genetics is shaping our subjectivities, and impacting on decision-making, treatment, and recovery in clinical contexts. We bring interdisciplinary perspectives from psychiatry, cognitive neuroscience, and the social studies of neuroscience to bear on the translational impacts of the neurosciences and epigenetics in new and expectant mothers in Quebec, Canada, as a case population. Our premise is that given the cultural authority of neuroscience, the application of findings to patients, practitioners, and lay users warrants careful analysis. This is particularly timely in view of important theoretical, methodological, and interpretive uncertainties in experimental methods and in the translation of neuroscience to societal applications, as the field moves to incorporate aspects of social and cultural context. While social theorists and historians have expressed significant concern about potentially reductive, individualistic, or pathologizing impacts on users, some have also overstated the transformative potential (Martin, 2010; Choudhury et al., 2012; Pickersgill, 2013). We explicitly seek to examine how consumers of these research translations understand, interpret, and are affected by epigenetic and neuroscientific information, rather than a focused discourse analysis of the translations themselves. This study provides an opportunity to bring nuance to this analysis through an understudied population of active consumers of this knowledge, and to examine how interpretations of brain science frame narratives about women's bodies and experience.

### The Medicalization of Pregnancy

Bodies as objects to be appraised, polished, promoted, protected, kept pristine as commodities, and assets have throughout history forced women to regard their own with suspicion. Early feminist writings push against any deterministic association between bodily characteristics, mind and its faculties, and social roles (Wollstonecraft, 1792 [1988]; Mill and Taylor, 1970). The female body has a history of social regulation whether as an object of desire, site of family control, or symbol of fertility, scrutinized, intervened, and controlled through formal and informal structures, narratives and images. Here, we are concerned with the role of biomedical science in the understanding and experience of the perinatal period among contemporary mothers and expectant mothers in biomedicalized societies. While biomedical science has a prominent role in lay approaches to motherhood, its role is not new and has its own history of management of women of reproductive age, during pregnancy and early motherhood. At the turn of the twentieth century, women in Western Europe and North America had minimal engagement with the medical profession over the course of their pregnancies (Al-Gailani and Davis, 2014). Social control of the female body was monitored through other cultural and religious institutions and channels. Within 100 years, the purview of science and medicine in human reproduction saw a striking evolution: the hospitalization of childbirth, The contraceptive Pill, prenatal vitamins, obstetric ultrasound, etc. (Al-Gailani and Davis, 2014). Some scholars argue that the transformation of pregnancy “from a natural event into a medical problem” (Seccombe, 1990, p. 181) has led to heightened scrutiny of “subjectively healthy populations” (Al-Gailani, 2014) and established new classes of patients and categories of disease (Al-Gailani, 2014). Though a deep treatment of this subject is beyond the scope of this manuscript, the historicization of the extension of biomedical authority, practice, and dominion into domains of women's preconception health and pregnancies contextualizes the current popularization and mobilization of contemporary biomedical approaches to optimizing fertility, infant health, and managing interventions.

Within the last few decades the field of epigenetics has shed new light on the mechanisms by which maternal environment influences outcomes in child development, and neuroscience findings indicate that experiences during “[neurobiologically] critical periods result in irreversible changes in brain function” (Nelson and Gabard-Durnam, 2020). The particular potency and reach of these new ways of thinking about pregnancy and early motherhood rest on a complex web of relations between the laboratory, journalism, policy makers, the vested interests of industry, and the affects, hopes, expectations, and social contexts of women of reproductive age. The specific forms and platforms of the translation of this research prevalent in the Euro-American context, the prevailing cultural rhetorics in circulation, the particular parties, and processes—all of which shape its bearing on women's perinatal experience—are unique to this moment. Yet, while the current actors and dynamics are specific to today, this phenomenon can be seen as part of a trend: a historical process of the increasing sphere of influence of biomedical science on life and self and the age-old utopic project of human improvement through scientific discovery and technological progress.

### Risk and the Making of New Norms

The study of the development of perinatal interventions demonstrates how both the identification of risk and the construal of risk are created in biomedicine and converge with social forces to make possible new ways of managing the (pre-)pregnant body. The prominence of medical regimes from diagnostic services to technological monitoring and intervention in the perinatal period has led many researchers to analyse the increase of “scientific motherhood” (Apple, 1995) and the production of new norms through biomedicine. For example, the mobilization of research linking folic acid to normal fetal development changed the relationship between the State, other actors, and pregnant women based on a moral imperative to mitigate risk and maximize optimization. The history of the now routine use of folate in pregnancy reveals how the emergence of new technologies in biomedicine afforded new ways of interpreting and delineating a “healthy” pregnancy (Al-Gailani, 2014). In the 1960s, for example, the development of microbiological assays of blood serum and its application to practice enabled the clinical study of megaloblastic anemias, which identified a “previously unknown problem”: without any clinical indications, a majority of women were mildly folate-deficient (Al-Gailani, 2014). Folic acid supplementation is now an imperative in biomedicalized societies—and globally exported as a biomedical norm—when trying to conceive, scaffolded by the interplay between scientific discovery, evolving medical practice, industry uptake, social and political interest, and popular messaging. In this way motherhood exemplifies a new and increasingly widespread way of thinking about health that combines a probabilistic logic of risk with the imperative to manage the future health of the body at the molecular level (Rose, 2009) through interventions in the present. The brain and genes of the mother and baby have become a contemporary site for this to play out.

### Plasticity, Intergenerational Transmission, and the Optimization of the Unborn Infant

In 2020, scientific research in the field of epigenetics exposes the phenomenon of intergenerational transmission of experience, further expanding the conception of the variables and necessary (windows of) interventions that constitute and engender a healthy pregnancy and optimal infant outcomes. These research bodies explore distinct temporal windows: epigenetic effects related to events or environments that precede pregnancy, occur during pregnancy, or during the postpartum period, where the plastic infant brain may also be affected by non-epigenetic means during critical periods of development. The plasticity of the maternal brain has also been the subject of inquiry both during and post pregnancy. Today, it is as if women are “eternally pre-pregnant” (Meloni, 2016, p. 217). New interpretations of epigenetics research not only have implications for risk management for the pregnant mother and unborn infant but also for the *potential* health of future generations. The transmission of traits across generation has long been conceived as the inheritance of genomic information, but recent research suggests that lived experience may be inherited through epigenetic mechanisms. Epigenetic research in animals—with a smaller body of literature reporting human studies—has suggested that variables ranging from trauma (Yehuda et al., 2014) and maternal mental health (Meaney and Szyf, 2005; DeSocio, 2019) to environmental exposures (Takiguchi et al., 2003), metabolism, diet, and other lifestyle conditions (Parle-McDermott and Ozaki, 2011), to postnatal maternal care (Bagot et al., 2012) have a bearing on cognition of the child. New neuroscience and epigenetics have been thus implicated not only in the management of preconception and pregnancy health of the mother but also in the optimization of the unborn infant. This is premised on pervasive messaging about neuroplasticity, or the impressionability of the developing brain. Specifically, cognitive neuroscience research on early childhood brain development points to critical windows of infant brain plasticity: the particular structural malleability and concurrent sensitivity to environmental stimuli confer particular potential for enhancement or vulnerability to affronts (Hess, 1976; Greenough et al., 1987; Black et al., 1998; Knudsen, 2004). It also points to changes in the maternal brain brought about by pregnancy and birth (Hoekzema et al., 2017; Barba-Müller et al., 2019) that may “not merely [be] adaptive, [but] likely confer a vulnerability for the development of mental disorders” (Barba-Müller et al., 2019). As Wastell and White (2017) write, “If brains can be damaged or boosted, should we not be boosting them or preventing the damage?” As scholars have already documented, the materiality of the plastic brain bears strongly on the popular imagination: the possibilities to influence developmental trajectories, reverse historical processes, or enhance/protect mental health by working on tangible cellular processes, that are visible at a macro-level through mesmerizing neuroimagery, is widely incorporated into clinical settings, public health messaging and popular science (Choudhury et al., 2012; Pitts-Taylor, 2016; Rees, 2016). Epigenetic science has already shaped policy and can be found referenced across a wide variety of cultural locales. Innovations in epigenetics and neuroplasticity related to mother-infant interactions have been of enormous interest to the media and public, with the Internet constituting a highly active space for engagement and cultural prosumption (Toffler, 1980) of translations of said research.

### Translational Impacts of Epigenetics and Neuroscience of Pregnancy and Motherhood

Popular media coverage plays a powerful role in the translation, reception, conciliation, and comprehension of science in the public sphere. “Traditional” media forms—including magazines, newspapers, radio, and television—that controlled a unidirectional flow of information to the public sector now exist in a broader ecosystem of platforms that support two-directional sharing of rhetoric, ideas, and information where audiences not only consume but also construct media content (O'Connor and Joffe, 2013, 2014) including for-profit company blogs, Instagram, YouTube, etc. A Google search of “epigenetics” surfaces a top hit—whatisepigenetics.com^1^—an alleged educational site by epigenetic biotechnology company EpiGentek, to “bring the science of epigenetics to the forefront of everyday life.” It contains over two dozen blog posts and claims to “translate” epigenetic research related to pregnancy to the lay public.

In 2016, Nature Neuroscience published, “*Pregnancy leads to long-lasting changes in human brain structure” (2017*) reporting that pregnancy was associated with reductions in gray matter volume. Popular UK online platform, motherandbaby.co.uk, centered on pregnancy and motherhood recommends “Top brain training apps to combat baby brain” and #MomBrain podcast launched in 2018 (Walling, 2018) with 119 episodes available through Apple Podcasts, Spotify, and other players. Headlines from *Science Magazine*, “Pregnancy resculpts women's brains for at least 2 years” (Wadman, 2016), *Parents* “Mommy Brain: Yes, It's a Thing” (Lucia, 2018), *Scientific American, “*Does “Pregnancy Brain” Exist?” (Does “Pregnancy Brain” Exist?, 2016), *Independent's* “Pregnancy really does cause ‘baby brain’, new research finds” (Young, 2018), and Instagram hashtags like #mombrain (appended to 97.4k posts), #pregnancybrain (31.3k posts), #babybrain (48.6k posts), #ppd (287k posts), reflect the exceptional public interest in brain changes over the course of the prenatal and postpartum period, the myriad actors and spaces involved in the prosumption of these bio-cultural narratives, and the influence of the biomedical in the realm of the subjective: experiences of pregnancy and motherhood framed as expressions of impacting and shaping “brainhood” (Vidal, 2009), the neurobiological recasting of personhood.

Over the course of a few decades, the neurosciences have come to occupy a significant place in individual and public life. Scholarly attention to this phenomenon has highlighted a contemporary fetishizing of brain images (Vidal and Ortega, 2017), the popular fixation on the brain, the blossoming of neuro-prefixes—such as neuro-education, neuro-psychoanalysis, neuro-aethetics—and increasingly common prioritization of the neuroscientific lens on phenomena once the purview of other disciplines of thought (Vidal, 2009; Rose and Abi-Rached, 2013). The primacy of the “neuro” in culture has led to assertions that we experience ourselves and lifeworlds in increasingly neurological as opposed to psychological or internal impressions (Ortega and Vidal, 2007): it is argued that we are more and more “cerebral subjects” (Ortega and Vidal, 2007) or “neurochemical selves” (Rose, 2003).

The dynamic and interactionist nature of the burgeoning new media landscape warrants increased exploration of public engagement with science across media platforms and increased scrutiny of the potentially unforeseen ethical and psychological implications of dialogue in these spheres. Further empirical analysis can also help to understand the cultural appeal of neuroscience and epigenetics.

The translations of biomedical information about pregnancy and motherhood in brain-centric idioms like “mombrain,” “pregnancy brain,” or “postnatal depletion” to narratives around maternal epigenetics—the impact of an organic Atlantic salmon roe diet (foundmyfitness, 2017) and the cigarettes one's partner smoked as a teenager (Kirkpatrick, 2016) on their child's cognition—have implications for the expectations, reference points, and self-imposed regimens for women during their pregnancies. Little to date, is known about how findings in these particular subject areas are interpreted by various publics. Empirical research has corroborated the distortions that occur when neuroscientific information permeates the public sphere (O'Connor et al., 2012) and critical neuroscience research has documented how health recommendations acquire scientific authority through references to the brain (Choudhury and Slaby, 2012). Interpretations are influential factors in an individual's psychological and physiological reality. So far, there is a gap in the literature assessing how the mobilization of brain and genetic data to frame motherhood is affecting women's choices and self-understanding.

In this study, we set out to address this gap by exploring how translations of neuroscientific and epigenetic information in the form of “epigenetic imaginaries,” (Jasanoff and Kim, 2009; Meloni and Testa, 2014), impact the experiences, attitudes, and mental states of women during the perinatal period. In this paper, we present results from focus group conversations with expectant and new mothers. Our focus group interview guide was informed by a prior familiarization with a range of diverse actors and outlets where epigenetic and neuroscientific translations are taking shape.

### Objectives

Based on our analysis of existing literature on the role of genetics and the “neuro” in contemporary biomedicalized culture, we were led by the broad question of how the primacy of the “neuro” in contemporary North American society affects women's subjective experience and understanding of their pregnancies and motherhood. To explore this, we approached the online material and focus groups led by questions such as: What does it feel like to engage with translations of epigenetic research? Is the take-away message from epigenetic research one of fixity or flexibility, control or lack thereof? How are women responding to or making sense of these translations? How do they relate to and feel about the cultural belief that pregnancy and motherhood changes the brain? To what extent is this brain-based explanation a liberating development or grounds for stigmatization? To what degree does “pregnancy brain” reframe expectations of competence or capability during and after pregnancy? What might the increasing prevalence and popularization of brain-based explanations indicate about the role of neuroscientific “proof” in the legitimization of women's experiences during the pre/postpartum period? Our overarching goal is to examine the functions of epigenetic and neuroscientific vocabularies and metaphors among a population who are frequently exposed to these ideas. This research was conducted during the COVID-19 pandemic. The specific context of the pandemic likely adds layers of complexity that may have intensified attention, shape awareness and affective experience of translations of these bodies of knowledge.

## Materials and Methods

We conducted three focus groups with pregnant women and new mothers. The aims of the focus group were to examine (1) how knowledge translation of epigenetics and neuroscience impacts women's decision-making and experience of the perinatal period; (2) the impact of this engagement on women's wellbeing and self-image.

The focus groups' semi-structured interview guide was developed against the backdrop of insights gained from an immersive background scoping study of online sources of biomedical translations that provided a foundational overview of where and how these bodies of knowledge emerge in public discourse. Given that the Internet-mediated world is a space of fervent exchange and debate around pregnancy, birth, and the female body for contemporary women, we sought to discern predominant narratives and dynamics online. Box 1 offers examples of online content that provide a window into the material that women can encounter online and provide added context for the participants' narratives that specifically mention Internet content.

Box 1 **Examples of Neuroscientific and Epigenetic Translations Online**

The following are four examples of online content related to epigenetic and neuroplasticity research that women may encounter. These examples do not represent the breadth and depth of digital translations of these bodies of research, but are illustrative nonetheless:

- A post in November, 2020 by a public Instagram profile reads “I used to have functioning brain cells, but I traded them in for children,” the text super-imposed on an illustrated image of a woman holding two children. The image's caption includes the following:
  “I read something the other day where a mom warmed up a plate of food, walked into the living room, sat down and thought—I'm hungry I should make something to eat. I don't know how many times I've walked into a room to do something and then forgot as soon as I entered◴♀◴ Seriously anyone else feel this way?!?
  #mombrain #itsarealthing #iusedtohaveagreatmemory #momoftwo #mombrainisreal #girlmom #boymom #lovemykids”
- A VeryWellFamily.com 2020 article—reportedly written by healthcare professionals and fact-checked (Verywell Family, 2019)—on “Mommy Brain” begins with the following conceit:
  “*Do you ever walk into a room only to forget why you went in there? Have you ever been searching frantically for your cell phone or your keys, only to find that they are in your hand? Or maybe you call your dishwasher the washing machine or blank out on the names of your coworkers. If you are experiencing any of these things, it is likely that you have “mommy brain.”*
  *Even though “mommy brain” may sound like a fictional condition or a convenient excuse for forgetfulness, it is actually a true condition backed up by science. In fact, research shows that a mother's brain is impacted by having children, sometimes in long-lasting ways*.
  *For instance, a study by the University of British Columbia demonstrated that motherhood has a permanent impact on your cognitive function*.
  *Meanwhile, a study in Nature Neuroscience found that even two years after pregnancy, women had gray matter brain changes. These changes took place in regions involving social cognition or the ability to feel empathy for another person. In other words, some subtle aspects of memory are sacrificed to enhance other areas of cognition (Gordon*, *2020**).”*
- A YouTube video titled “*Epigenetics”* published on January 22, 2012 by the YouTube channel SciShow. At the time of writing this video was the number one search result for a search query of “epigenetics” on the YouTube search function—filtering by view count—with 2,299,856 views; SciShow had (6.53 M subscribers). The video length is 9 min and 29 s. The transcribed audio from minute 5:32–6:09 is as follows:
  “*And it just so happens that the more they study this, the more it looks like bad epigenetic information is being passed from generation to generation. And this is a whole new way to think about how we pass information between generations*.
  *Your grandmother was making dietary decisions that affect you today. As we experience all these new strange epidemics—diabetes, autoimmune disorders, cancers—that weren't appearing in previous generations, it's starting to look like these may be caused by epigenetic information passed down from our parents*.
  I know! It's such an unbelievable buzzkill! There is no point in our lives when we can do anything without guilt anymore!”
  At the time of writing, this video has 4,749 Comments. When sorted by “Top Comments,” the text of the first two comments are:
  1: “I actually think this is uplifting rather than depressing. If you choose to have offspring, you can make decisions now that give your descendants a potentially better life. Exercise regularly and eat right? Your kids might be more likely to do that, regardless of your original genetics.”
  2: “DAMNIT GRANDMA”
- The first three paragraphs of a blog post published by whatisepigenetics.com titled, “5 Ways You Might Epigenetically Boost Your Child's Health Before Birth,” published January 29, 2018.
  “*When the Twin Towers came down in 2001, it was one of the most shocking moments in human history. This brazen act of terror traumatized an entire population. For those who lost friends, family, and acquaintances in the tragedy, it was an enormous cause of stress, grief and general departure from a normal state of being*.
  *Among the affected, many were pregnant women*—*some of whom developed PTSD after the incident. As reported in The Journal of Clinical Endocrinology and Metabolism, when these women gave birth, there were certain peculiar effects observed in the children. The children who had mothers with PTSD were born with lower levels of cortisol, whi-ch is known as the stress hormone. In addition, their responses to stress-inducing stimuli in their environment were dysfunctional*.
  Although none of these kids had witnessed the horror themselves, their biochemistry reacted as though they had. This wasn't some random coincidence. It was a demonstration of the power of epigenetics.”

Ethics approval was obtained and sanctioned by the Institutional Review Board of McGill University (IRB Study Number A10-B60-19B).

### Focus Groups

#### Recruitment

Participants were recruited through two local organizations in Montréal providing perinatal services and by posting the opportunity to a local Google Group for parents. Recruitment occurred between July and November 2020. The total number of study participants was reached through the processes of purposeful and snowball sampling. Recruitment sites were chosen to recruit as demographically diverse a sample as possible so that group composition reflected a range of vocations, socio-economic statuses, ethnicities, educational backgrounds, and ages. Women who had already given birth were required to have a child under the age of 5-years-old. Participants were informed of the study objectives, focus group process, and data protection prior to participation. Informed consent was secured in writing and participants indicated whether they preferred not to have their name associated with their comments. Women could choose to rescind their participation at any point without explanation. Three women initially signed up to participate but were unable to attend the focus group due to scheduling conflicts.

#### Focus Group Guidelines and Process

Three separate focus groups were held with a total of 17 participants: the first and second group comprised six women and the third group, five. Discussion was steered by a semi-structured interview guide developed by the research team. The interview questions were designed to stimulate discussion by providing a starting point for respondents to contribute further statements on the subject. Questions were not asked verbatim across groups nor was there a strict chronology in delivering specific questions across groups. The questions were posed so that the interviewer could probe particular subject areas that arose as thematically pertinent and direct the conversations to foster a degree of topical consistency across the three groups, as fitting. Participants discussed questions based on their own personal experiences and point of view.

The questions were divided into eight themes: information sources about pregnancy and birth; social media platforms; biomedicine; genetics/epigenetics and motherhood; neuroscience, the brain and motherhood; expectation; birth; motherhood and support systems.

Questions included (but were not limited to):

- Queries about general types of pregnancy and motherhood content that participants engaged with during the perinatal period and from where this information was sourced;
  e.g., “*Where have you learned about what to expect during pregnancy?”*
- Whether and under what contexts participants sought out biomedical information;
  e.g., “Have you come across or actively searched for medical or scientific information about pregnancy, birth and motherhood? For what aspects of your pregnancy do you look to medical or scientific literature to learn about? (Or do you not engage very much with medical or scientific perspectives on pregnancy?)”
- Specifically; in each group, participants were asked whether they were familiar with the term “epigenetics” and for those who did not recognize the term, a few popular headlines related to epigenetics were read to the group for reference. These particular headlines were selected as complements given they reflect diversity across several domains: (a) the degree of certainty communicated through language choice: “*permanently* influences,” “*may* raise,” “*could* pass on”; (b) the particular topical focus of article: diet, smoking, stress, exercise; (c) inclusion of one paternal study; (d) inclusion of a non-traditional media outlet, “whatisepigenetics.com” which—for the first author—appears within the top five Google search results using the term “epigenetics” and top two search results using the query “epigenetics pregnancy.” Listed below are the headlines which were selected.
  “*Is the term “epigenetics” familiar to you? If yes, where and how have you interacted with it/learned about it?”*
  *If not, here are some popular press headlines. What are your initial reactions to this information?”*
  - *BBC: “Pre-pregnancy diet permanently influences baby's DNA” (Briggs*, *2014**)*
  - *Reuters: “Young male smokers may raise obesity risk in their future sons” (Earls*, *2010**)*
  - *NYTimes: “Inheriting Stress” (Gaisler-Salomon*, *2014**)*
  - whatisepigenetics.com*: “Parents Who Exercise Could Epigenetically Pass on Heightened Learning Ability to Their Children” (Kirkpatrick*, *2018**)*
- Similarly, participants were asked whether they had engaged with any neuroscientific content during the perinatal period, and specifically whether terms like “mombrain” or “pregnancy brain” were familiar to them.
  e.g “Have you encountered or heard of the term “mom brain”? If so, where have you learned about it and what does it mean to you? If not, what might it indicate?”

The first focus group was moderated by the first and last author, who both—to avoid influence (Krueger, 1998; Krueger and Casey, 2000)—refrained from participating in the discussion except to ask for clarification or further explanation and elaboration. Participants spoke on their own initiative and engaged with each other's responses. Focus groups lasted between two and two-and-a-half hours.

The methodology had to be adapted to the evolving COVID-19 pandemic restrictions. The first focus group was conducted in-person following the social distancing measures in Montréal at the time. It was held in a non-public space with a comfortable atmosphere. The first and last authors were present, as was a local birth advocate and postpartum doula. The presence of a doula for this first group discussion was intended to ensure comfort and security for participants, and to hold space for any mention of emotional difficulty. Due to changes in COVID-19 pandemic regulations, the following two groups were held over video conferencing platform (Zoom). This allowed for participants to join remotely from the comfort of their own homes. The Zoom groups were moderated by the first author only. Anticipating the pragmatic challenges for group rapport presented by a digital focus group, the last author and doula refrained from participating. The rational was to keep the group as small as possible, to enable the intimacy required for the participants to comfortably share their experiences. Though we decided to forego the presence of the doula in the Zoom sessions, participants were given the option to speak with her if they felt they needed to debrief. Conversation was felt to reach a comparable degree of intimacy across in-person and remotely orchestrated groups. On Zoom, however, though participants shared equally personal narratives to the first in-person group, discussion took on more of a turn-based form. In person, participants were more likely to prompt or interrupt each other in echoes of agreement, difference of opinion, or clarification. On Zoom, participants tended to mute their audio while others were speaking and there was often a pause in between speakers. All focus groups were audio recorded and transcribed; names were pseudonymized in transcription. Field notes of initial impressions about pertinent themes were made after each focus group.

### Data Analysis

Focus group data were coded manually on paper and digitally. Thematic analysis was conducted by the first author; broad themes were identified and discussed among the authors. Any discrepancies that arose were resolved by incorporating the perspective of the last author. The analysis was guided by the overriding research questions, an awareness of a diversity of online sites and forms of research translation afforded by the scoping study of online sources of biomedical translations and the resulting awareness of the translation narratives circulating online, and the questions and discussion during the focus groups. Thematic analysis consisted of searching across the corpus of data and within individual focus group data sets.

Themes emerged in the data set vis à vis focus group participant responses to the prompts that guided the discussion. In this sense, themes emerged not only for their prevalence and relevance across data sets (at the level of individual participants and at the group level, across three separate focus groups) but also based on the emotional quality of certain content over others. The first categorization of transcribed texts resulted in an initial grouping of themes that was further refined through an iterative process with an increasingly interpretative lens.

Our analysis of the focus group data took two stages. Transcripts were read multiple times, and studied to identify the themes that related directly to our research questions. A second inductive approach was also employed by the first author to locate additional salient themes within the data, and discussed amongst the authors. Our thematic analysis was theoretical in nature and largely at the latent level: driven by the analytic interest in specific issues and concerned with the identification and examination of base assumptions or perceptions that influence the semantic content (Braun and Clarke, 2006). The categories of themes presented in our results section reflect semantic thematizing i.e., navigating biomedical and cultural perinatal information on the Internet and latent thematizing i.e., participants' narratives that provide evidence to certain psychological phenomena or reflect evidence of a particular cognitive mechanism at work, such as looping effects, that directly speak to the potential impacts of engagement with translations of biomedical research on the perinatal period.

Thematic analysis was contextualist, positioned between the poles of an essentialist or constructionist theoretical method: we sought to “reflect [the] “reality” of participants while also “unpick or unravel the surface of ‘reality’” (Braun and Clarke, 2006).

The focus groups were lagged, separated by at least 1 month, which allowed for extended reflection between discussions.

## Results

The results include a demographic overview of our sample and the presentation of the three themes that emerged from our focus group data. The analysis and results presented here speak to the focus group data set reflected in Tables 1, 2.

**Table 1:** Sample demographics.

**Demographic categories**	**Frequency**
**GENDER IDENTITY**	
Woman	3
Woman (she/her)	1
Female	10
She/Her (female)	1
Straight female	1
Cis gendered woman	1
**AGE**	
26	1
33	3
35	4
36	1
37	3
40	3
41	1
42	1
**MARITAL STATUS**	
Single	3
Married	10
Separated	1
Divorced	0
Conjoint	3
**HOUSEHOLD INCOME**	
25–50k	3
50–100k	7
100–200k	4
Over 200k	2
Preferred not to disclose	1
**ETHNIC IDENTITY**	
White	3
Caucasian	2
Canadian of Italian descent	1
Italian/Canadian	1
White, British, Jewish with immigrant parents	1
White Newfoundlander	1
Caucasian/French Canadian/Irish Canadian	1
Ukrainian	1
Latin American	2
Brazilian	1
Chilean/Latin American	1
Black	1
Preferred not to disclose	1

**Table 2:** Focus group discussion narrative themes.

**Theme 1. A kind of brain**	
**1.1 “Mombrain” brain as validating subjective experiences**	
**1.1.1 Alice:**	*But this most recent pregnancy, I was struggling a lot with stress and brain fog. Really feeling like I'd lost my edge. I'm not even me. Everything is like a soup. I was looking for academic research, “what are the effects of high levels of estrogen on cognition in women.”… Looking for published research about what is there out there that might explain my subjective experience in terms of a scientific possible explanation...There's a bunch of stuff online that's kind of like, “mommy brain's not real...” It's real. It's absolutely real.I can't think at all. And I feel like this is where I end up going. But I'm like, this has impact on my career. This has impact on my learning. This is an actual phenomenon. Not just women complaining. You know, not just women being lazy or whatever. But like an actual phenomenon that I can find no mention of in anything besides like pop reporting and that's why I started looking for, ‘is there any actual research out there about estrogen levels and cognition?’ That would legitimize what I'm subjectively feeling*.
**1.1.2. Gabriella:**	*I think [the neuroscientific terms] justifies why you do things. And then you can explain it to people, yeah that's scientific. (Laughs) Like it's not just a crazy me thing, it's an actual thing that happens to most women who are pregnant*.
**1.1.3. Hailey:**	*I do sometimes blame hormones for something which clearly originate the brain, but it's also another system. We like to call women hormonal and it can be negative, but at the same time sometimes I like to attribute it to a process that's happening within my body. Especially say like, postpartum, you have this adrenaline for a few weeks. And then, depletion, the baby blues or whatever. We kind of cry out of... I mean, I clearly want to attribute that to this hormonal shift that's happening in my body and not the fact that I can't control my emotions. And so I guess I use what works for me when I want it to...I feel like I legitimize certain things based on how I want to. It's not just, I can't control it. It's because there are these things happening in my brain and my body and learning about it can help to sort of think like, okay, oh, this is normal… Sometimes I want to use it for my benefit. Like I make an error in sending a letter or something like that. Well, I use it to my benefit when it works out, like a horoscope. When it doesn't work out, I don't like it*.
**1.2 “Mombrain” as stigmatizing**	
**1.2.1. Louise**	*I feel like we hear a lot about [mombrain] in popular culture. I clicked on something on the Internet the other day, I think it was something on PET scans [inaudible] like, there's less activity in the hippocampus in women who've given birth for some number of years afterwards. I've heard of things like that. So I know there might be some evidence to it. But still, like, I don't like the concept in general because I feel like for me, I went back [to work] like six months postpartum and I had exams to take and stuff like that. I kind of felt that the fact that this is a popular concept in media and the culture in general, I feel that I hope it doesn't contribute to people's impression of me at work when I'm back after having a baby, et cetera. So, in that sense like I didn't like it so much and I didn't find it to be true personally. Yes, of course, like if I didn't sleep well, then I was tired the next day, but I took like exams and stuff, maybe like a year-and-a-half postpartum and I did just as well as I had done on previous ones, so I feel personally, I was fine. It's not the greatest concept if it's going to discourage people from either doing things at work or if it's going to affect other people's perceptions of them. Just pretending it doesn't [occur] seems okay for me. So that's what I'm going to do*.
**1.2.2. Beatriz**	*I never felt someone was holding [mombrain] against me or saying, 'Oh, she was not as good because of that' or something. No. I never felt it. But I felt it myself, inside. I felt I was not being good enough. I feel, I forget. I put more pressure on myself because, Oh my God, why didn't I forget, is because of my mom brain? And I am like anxiously looking for [my memory] to go away again*.
**1.2.3. Louise**	*When the term brain fog is used, it sounds like it's something that's less correctable or you can't change it as much versus if you say, 'Oh, it's because I'm tired and I'm pregnant', well, there's an end to the pregnancy and you're not gonna be tired if your baby starts sleeping better. If you say that there's like a permanent or at least long lasting change; that pregnancy and being postpartum causes cognitive changes in the long run over several years, then I find it becomes problematic because when you return to work and there are expectations regarding your performance, you might feel as though if other people believe in this concept. The idea that there's brain fog makes it sound like you might be less competent versus if you say it's like hormonal changes or you're sleep deprived or it's the pregnancy: those are all things that come to an end fairly quickly. So they can't be used as a longterm performance problem. Because they specifically write an article that talked about there being changes that lasted at least up to three years based on their follow-up period in the study. I have experienced periods, especially like early postpartum when sleep deprivation is very prominent, I feel like I have a certain amount of brain fog, but I guess it's just that the idea that there's really some lasting change that has a negative effect is less appealing*.
**1.2.4 Zoey**	*But I, what I find frustrating [is that] there's this trope for so long about women can't be leaders because of our menstruation because when we have PMS, like we're crazy and wild. I think mom brain fits into the same thing where [the] narrative is compared against [a] male standard. Publicly, it's not like, wow, women are so powerful when they're in ovulation, they can be incredibly outgoing and charismatic and creative when they're in PMS, they're incredibly sensitive. The veil thins between the conscious and the unconscious, and we're in this period of being sort of shamanic beings. And so I think during pregnancy, there's this huge spiritual aspect that is totally ignored and repressed. And so the value and the power and the capacity for pregnant women to play this incredible role in society is downplayed. And instead, what, what gets projected out is, ah look she becomes a shitty employee... So I think it's just this patriarchal standard and it doesn't serve us. And it's kind of like pinpointing, like using against us what, you know, never is talked about in a meaningful way: men, because they have so much testosterone should not be leaders because they have a tendency towards war and aggression*.
**1.2.5 Maya**	*Around the brain fog first: the balancing of the narrative for me is the important thing. Cause it's like a big part for me. Doesn't like these hashtags, you know, hashtag brain fog, hashtag mom brain partially because of the impact that a lot of this stuff had on me in terms of like my work, you know, and the unspoken sense of not being as competent: obviously people not really being allowed to say so, but it's kinda there, you know, and there isn't exactly space for it. Right. So I just feel this real tension between wanting to acknowledge that this is a very real thing, right. Where I'm just like, ‘my memory was wasn't as good’, you know, like there's many ways in which I wasn't as capable in terms of being productive in a sort of capitalist productive way. I was very more creative and more able to do certain things, but definitely less able to do others*.
**1.2.6 Phoebe:**	*I've heard about pregnancy brain and stuff. Am I like just pointless to them once I become pregnant? And then eventually have a kid? That's like a huge thing that I'm dealing with. I'm trying to over-perform now so that I can be like, 'I can do two things at once'. I want to leave work on a high note and just like, remind them that I'm like still a good employee. So a lot of that pregnancy brain, mumbrain is a huge thing, I think, um, in terms of my career and how I think about work specifically, like, I don't, that's where I see like the measure for failure*.
**1.2.7 Nina**	*I think like the use of the word brain fog, like, you know, in some cases maybe it feels accurate, but like the universal use of it is probably because we have a tendency to like blame things on women and mothers in particular. So like to make it about the mother's brain is not really fair. You know, you might just be tired. I worked really a lot, like more than I probably should have the whole time I was pregnant up until the last, like three or four weeks when I took some time off. But I didn't find that there was a problem with my brain. I found that I was tired and I would take small naps in the afternoon*.
**Theme 2: The looping effects of biomedical narratives**	
**2.1 Ripples of knowledge**	
**2.1.1 Louise:**	*Like, you have your genes and your genes are supposed to be set in stone, except that there are environmental things that can cause changes in the gene that persist over the longterm. So like, example, what I've heard of is like, Oh, if there's stress in pregnancy, like COVID, like with my daughter. So I heard about that... like a big environmental event or multiple little ones that can change your genes, well they might remain changed that way down the road for many years and maybe even passed on for the next generation. Which is I think where the interest in pregnancy comes from... pregnant women and stress and how it could negatively impact the baby...I think it was in regards to like pregnant women and like some natural disaster that had occurred like either a flood or a fire somewhere*.
**2.1.2. Nina**	*I read a paper one time about, people who lived in the Warsaw ghetto during the second world war. Uh, and there was like a lot of food shortage and there was some potential longterm effect on their descendants of like body mass*.
**2.1.3. Hailey**	*I think it was the ice storm. I was surprised that like a two week period could have such an impact. This pandemic is going to go on for much longer, like say the Warsaw example, I mean that's quite more distinct in time. I was also part of another research study that looks at stress in pregnancy during the pandemic. And I think they are interested to see different markers cause they're also now asking for like either a hair sample or something else. The one thing that worries me is the impact of stress during pregnancy*.
**2.1.4 Alice**	*I was actually worried about epigenetic effects in the baby. Worrying maybe that they would be more sensitive to stress or what have you. I wasn't worried about things like Down's Syndrome or developmental… and I wasn't particularly worried about preterm labor or anything like that even thought I know that high stress can be associated with preterm labor. For me personally I wasn't really worried about that. I was confident in my physical health while I was pregnant. I was mostly concerned about my mental health and any potential epigenetic effects that would have on the baby… I deliberately avoided all forms of literature about effects on babies of stress in mothers because I was maximum stressed*.
**2.1.5 Teresa**	*Well, I'm stressed out today because life is stressful. But I shouldn't be stressed cause that will hurt my baby. It ratchets up all of the stress that you're feeling.…there were some times that I was frightened and really angry and really unhappy and I was thinking I can't protect my baby from these feelings, from whatever's happening to me physiologically. So, I definitely did have those thoughts. What is the effect of this fight? This blowout? Me being frightened? Me being angry? Me being really hurt and I can't protect her from it*.
**2.1.6 Gabriella**	*I had so much trauma since January, my levels of cortisol were so elevated all the time and when I was working it was easier to be distracted by something so cortisol levels would come down but now my cortisol levels were so high all the time, all I could think about was, how is this going to affect her when she comes out? Right, because everybody tells you, you have to stay calm, you have to be so happy… I'm crying all the time, I'm loosing my mind, I don't know what's going on. And all I think about is, “cortisol is too high, I've gotta calm down.”*
**2.1.7 Charlotte**	*Just to add to what you were saying about “knowing” and actually being able to do… if you know it's better to eat a certain way or to do…I was on anti-anxiety medication for many years and the fear was this medication, is it going to impact my unborn baby? If I'm finding other ways to self medicate, is that going to impact my baby? So it was a lot of weighing whose mental health is going to be more important: mine during this pregnancy and the potential impact that it has on my child or should I be focusing more on the unknown and my child's development while I may suffer mentally during the pregnancy? So it was kind of a battle to know this is probably not best for me to be on medication, but at the same time if I'm not then this is not going to be a healthy pregnancy for me…*
**2.2 Ripples of risk and diagnosis**	
**2.2.1. Beatriz**	*You do get flooded with all kinds of scary things. The talk about postpartum depression: it's so needed. It is. And of course, you know, you need to be aware of it, but just talking about having it was giving me so much anxiety that I was like every 15 days seeing a doctor to prevent postpartum depression that I never would have in the first place. And honestly, the doctor, he was great, but it wasn't that that saved me, you know, like it just didn't happen with my body. So it does create needless anxiety. I was dealing with a lot of anxiety and I was hearing that having postpartum depression was gonna be a sure thing for me. My mom had it for me after birth. So I was like it's going to happen to me, I have it in my genetics. So I prepared. I was afraid of it. As a mom, everything you hear, you get so afraid. I would say that it's the news and everything that comes out of it. It's so sensationalist. As a mother hearing about epigenetics and all this sensation about it*...
**2.2.2. Zoey**	*Women are taught to have so much fear during pregnancy*
**2.2.3. Victoria**	*Most [stories of pregnancy] are not positive stories; I think in pregnancy and motherhood we need to see more positive birth stories. When I was in England, that was a very, very important discussion. There was a lot of groups to share positive birth stories you know, most of the times we get more into the negative and we of course can freak out. Positive stories are super important. I think if we could get a balance, you know, between positive and negative birth stories*...
**Theme 3: Imprints of past experience and the management of the future**	
**3.1 Translational trauma**	
**3.1.1 Maya**	*Um, similarly I heard something again, I don't know how verified it is. Someone sent me an article [about epigenetics]. I think it is that their experiences or traumas, this got imprinted on their DNA in some way. And that that gets passed down. And I remember being, first of all, it just seems so sci-fi that, really, it like sticks to your DNA, that experience? Then I got nervous cause I was like, Oh my God. Thinking about my grandmother's experiences. And then thinking about my own son and, and you know, my partner's mother and then my mother and just being like, I have no control over this, you know, they've been through so much, he's going to experience that on some level maybe*.
**3.1.2. Victoria**	*I think it also has a lot to do with the idea I was suggesting before that the brain is plastic. You can always change it, you know, in a positive or wrong way, but it can be changed...There's also a lot of negativity about epigenetics. We forget, or maybe we don't know much, but with epigenetics, we can also do positive things. Life gives us the chance to change it again and to make it right. I think it's positive to be aware of the concept to try to understand we can use it for positive*.
**3.1.3. Victoria**	“*I'm very familiar with neuroscience, especially now, with the kids getting older. I read a lot and most of it has to do with neuroscience and the way the brain is shaped and how the early years are super important...So the experiences you get are very, very important, especially in early years. Even though you don't have your dream birth or the best pregnancy, the thing is that you can change it, you can, you can always do better...I think it also has to do with the way you parent…little rats: one didn't have like the mother who would [care for her baby] mouse. They moved it with a mother who had [caring behavior] and that little rat with no genes to be caring when she became a mom, she was caring too. So the expression of the change of the gene suggests that we can change the way we help our kids...You know, you have every day to make it better and every day to achieve a positive experience with your kids*.
**3.1.4 Beatriz**	*They take a scientific paper, they take one piece of information, they make it a big headline. And then they talk about it like it was the end of your life. Your child is going to be abuser or is going to be a rapist because your grandfather was. It's like, it's they take it out of context and it creates so much anxiety. And it's like, no, you know, it's such a small thing. The body has so many protection mechanisms. That it's not because something happened in the past, they're doomed to happen again. So balancing that perspective with being in the middle of the feeling and receiving all that information, you know, it's kind of hard for me and I kind of forgot about my theory, forgot about what I knew. I forgot about the deeper analyses and inside me I was like, Oh my God. And I had to remind myself, no, I dyed my hair, but my baby is going to be fine. And my grandmother killed herself when she was 40, but I'm going to be fine. My baby's going to be fine. It's a lot of work. I find that it's a lot. It's intense*.
**3.1.5 Efe**	*I've heard the term epigenetics here and there. And so I had like a vague idea about it that, the things that you do in your life will have... you have power in influencing your genes. I'm an adopted person and I don't know anything about my family. I don't know anything about like my genetics. I kind of sort of felt like a blank slate. Not because I am, it's just the reason why I'm here in Canada was because of, you know, war in my country of origin. That's why I was, that's why I got adopted. That's why I'm here. So it's like, I know that there is a lot of, you know, trauma in my background. I'll just live my life and do the best I can. I don't know anything about [my background], so I do think about it, but the only thing that I can do is my best. So I'm not, I don't really want to like put too many ideas in my head because it's just like, we don't know. It's too up in the air for me. Like it's just very abstract*.
**3.1.6 Rosa**	*There was child abuse included in the list of things in the generations before me and me included. And I was very scared of, because I didn't understand. I thought it was more like you will end up by, um, attracting that to you because of the way you act or the way you relate to people. I never considered that it was in DNA. So I'm like, okay, how do I stop the child abuse? I'm very stressed and anxious about it. So I did go to a psychologist that is dedicated to children. And I'm like, okay. So how do I prevent my child from being in a situation like this?*
**3.2 Responsibilization of the mother-to-be**	
**3.2.1 Alice**	*Something that's so frustrating about that—whether it's epigenetics research or just like ‘eat well because it has an effect on the baby—sometimes that's accessible and sometimes it's not. Particularly the things that are out of an individual person's control. It made me angry at our society. This is ridiculous. It's like we have information telling us that having elevated cortisol levels and super high stress is absolutely associated with negative outcomes. But, there's no support for you. You have no job. Do the things. Go ahead. But, keep going and eat a fucking salad. I think particularly in the context of being a pregnant mother with an innocent, helpless human inside of me who I'm solely responsible for, it feels like a huge weight of responsibility*.
**3.2.2 Teresa**	*I think that there was some part of me that was very stubborn about resisting that kind of information because I felt like that it wasn't something that I should have to take on: that I should have to be worrying about every single thing I thought or felt or did. And so there was some part of me that was very rebellious that way. And then every once and a while I would get sucked in and it would cause me this terrible anxiety and I would have to go back and sit and think about what do I want, how do I feel? Do I feel healthy? Or in the cases where after my child was born I would look at her and go, ‘does she look happy, does she look healthy?’ Constantly trying to pull myself back to that because of this glut of information*.
**3.2.3 Teresa**	*The other frustration for me which is less personal, it's more social, was this information should be used to make structural changes to lessen stressors on people's lives...We seem to have this idea that regardless of the science whether it's positive things you can do or negative things you shouldn't do, it still places enormous expectation on individuals*.
**3.2.4 Zoey**	*There's a lot of moralizing that goes on around pregnancy*.
**3.2.5 Alice**	*When I think back to 15 years ago when I was pregnant with my first daughter—I don't talk about this much because I was trying to fit into mom society—I was 19 and I was pregnant and we lived in my car. And we kept trying to apply for welfare and they kept denying the application. And we were eating at the food bank…that does a hot lunch every day…so our whole life was going around in this broken ass uninsured car…that I couldn't get inspected cause we had no money. Go to one place to line up, get whatever they're serving. And it's mostly bread. And go to the other place for dinner and it's mostly bread. And you go to the food bank and they give you frozen expired yogurts that are all aspartame and granola bars that are all aspartame and like a two liter of Nestle Quick Powder and more bread and some pasta and a can of beans and then you're reading, “I need to be getting adequate nutrition” but if it's beyond your control to do that then it leaves a lot of stress on the individual without any societal support. Things that you can't change, wish you could, but are educated enough to know that they might have a negative effect on your child, it's infuriating to me*.
**3.2.6 Louise**	*I feel that there was somebody…who gave an interview to the press about like women, pregnant women and stress and how it could negatively impact the baby. Except that this article came out in like April or March maybe. And I was due in May and of course I had already been stressed due to the pandemic. Oh geez. It kind of sucks when it's something that happened and you have limited control over it. Cause I think I remember like the initial time I heard about [epigenetics], I think it was in regards to pregnant women and some natural disaster that had occurred like either a flood or a fire somewhere. So that seems like very far away to me when I heard first heard about it. Cause I was like, Oh, you know, that's interesting. But you know, of course: pandemic. So I got my own little taste of that with this one*.

### Sample Demographics

Our sample consisted of a total of 17 women. Four participants were pregnant at the time of discussion. All participants hailed from Montréal and the surrounding area, representing eleven different neighborhoods. The mean age of participants was 36. Listed occupation spanned a variety of industries and positions represented various rungs of institutional hierarchies (e.g., medical resident, operations manager, etc.). See Table 1.

### Focus Group Discussion Narrative Themes

On the basis of focus group material, three main thematic areas were identified: (1) A kind of brain (Table 2.1); (2) The looping effects of biomedical narratives (Table 2.2); (3) Imprints of past experience and the management of the future (Table 2.3). The results will be summarized in brief and elaborated upon in greater detail.

*Theme 1: “A kind of brain”* (Table 2.1) captures women's perspectives on the concept of “mombrain” or “pregnancy brain.” This theme encompasses women's reflections on this “kind” of brain, discussing the extent to which this concept was validating or stigmatizing and how its popularization impacted their experience of pregnancy and motherhood. For some participants, the notion of “mombrain” provided the legitimization of and justification for their subjective experience of e.g., memory lapses or forgetfulness—the phenomenology subsumed under this term—during the perinatal period. For other participants, “mombrain” created expectations of incompetence and was the cause of worry. The brain-based explanation was considered to render the phenomenological experience more serious, permanent, and without obvious solutions. Alternative explanatory models were proposed e.g., sleep deprivation and hormonal shifts. Some interlocutors felt that the interpretation of biological difference aids a societal construction of female limitation.

*Theme 2: “The looping effects of biomedical narratives”* (Table 2.2) addresses several impacts of biomedical narratives on the expectations and the experience of the perinatal period. Women discussed their engagements with translations of epigenetics and neuroscience as anxiety-inducing. Participant narratives revealed that consumption of knowledge translations of epigenetic research increased scrutiny and awareness of mental states, creating distress around the current or anticipated presence of stress, anxiety, and depression and the potential impact on the baby. This theme reflects that engagements with epigenetic research translations have the potential to precipitate and perpetuate distress inducing categorical loops and bioloops (Hacking, 2000).

*Theme 3: “Imprints of past experience and the management of the future”* (Table 2.3) is linked to the concepts of epigenetic inheritance, permanence and plasticity and the societal responsibilization of the mother/-to-be. The engagement with epigenetic research translations discussing transmission of trauma at the layer of the epigenome left some women with a feeling of incapacity to control or act upon past experience. This was a source of distress. Other women discussed the concept of plasticity as proof of their ability to repair and enhance, conferring a sense of agency. This potential ability, agency and biological flexibility, for some implied an overwhelming degree of responsibility and blame-ability. A number of participants voiced frustration that translations of epigenetic and neuroscientific study supported an imperative for them to monitor their bodies to mitigate risks and promote optimization of their children.

## Results

Our findings cast light on how engagement with translations of epigenetic and neuroscientific research impacted women's perinatal experience, wellbeing, and self-construal. At best, the narratives and framings of translated scientific research can alleviate feelings of guilt and stigma. At worst, they can reinforce stigma and evidence suggests that data is being mobilized to create stigma against women from disenfranchised backgrounds, with echoes of eugenics from decades past. (Richardson et al., 2014; Lappé, 2016). The neuroscience gives rise to a new “kind of brain”: the “pregnant brain” or “mombrain.” This “kind of brain” for some serves to legitimize subjective experiences of change and challenges during the perinatal period for others this biologization increases/results in stigmatization of women of childbearing age. The authority of neuroscience and epigenetics in our society confers a high status of truth to this knowledge. Women's narratives attest to the epistemic status of these forms of evidence to bring about perpetuating cycles of distress. Interpretations of epigenetic science revealed tensions between perceptions of determinism, biological damage, lack of agency, and potential pressure experienced by narratives of plasticity and opportunity for optimization. In line with existing analyses in the literature, the translations of these knowledges also confer responsibilization of the individual and create imperatives of self-monitoring.

### Theme 1: A Kind of Brain

Respondents interpreted the popular science and public health literature on neuroscience and epigenetics as evidence that points to a particular “kind of brain,” a configuration of the brain's structure and function specific to pregnancy and early motherhood.

#### Mombrain as Validating Subjective Experience: “It's Not Just a Crazy Me Thing, It's an Actual Thing”

On December 19th of 2016, Nature Neuroscience published a paper, “Pregnancy leads to long-lasting changes in human brain structure” (Hoekzema et al., 2017), that was immediately picked up by major traditional news outlets like *The Scientific American, Science Magazine, The New York Times*, all communicating with slightly different words, the “take-away” from the study: “Pregnancy Causes Lasting Changes in a Woman's Brain: New mothers showed evidence of neural remodeling up to two years after giving birth” (Caruso, 2016). This paper reported significant pre- and post-birth reductions in gray matter volume of brain regions including several cortical areas in addition to the hypothalamus, amygdala, nucleus accumbens, and hippocampus (Hoekzema et al., 2017). Although neuroscientific research on cognitive performance or memory decline (during pregnancy) remains largely inconclusive (Barha and Galea, 2017; Duarte-Guterman et al., 2019) its uptake in lay media and its ascription to increasingly common notions of “pregnancy brain,” “mombrain,” or “brain fog” does not always reflect this. A New York Times piece proposes cognitive deficit or memory loss as an attunement to infant needs: “*It may be that some subtle aspects of memory are sacrificed to enhance other areas of cognition”* (Sacks, 2018). WebMD's treatment of the subject follows the same formula: “*It has been postulated that, from an evolutionary standpoint, this memory impairment may be helpful so that women will forget about other stuff and focus on caring for the child”* (Mann, 2014). Examples of the notion of a trade-off between cognitive function and having children can be found across the social media sphere: posts by pregnant women and new mothers on Instagram incorporate this rhetoric into their communications, performances, and self-construals (Box 1).

The majority of women in our sample were familiar with the terms “pregnancy brain,” “mombrain,” and “brainfog.” Discussion highlighted two dominant reactions to these terms that revealed tensions between women's personal relationship to the phenomenon and their feelings about its implications in society. A number of women fervently asserted that forgetfulness, memory lapses, or absentmindedness during the perinatal period—the phenomenology subsumed under the concept of mombrain—are not imagined phenomena: “*mombrain is real*” (Table 2: 1.1.1). In their minds, they were not as capable during pregnancy and motherhood as they had been before. To these women, brain research played a legitimizing role. Their forgetfulness could be justified by the brain; public dialogue substantiated the prevalence of this subjective experience and provided authoritative proof of its realness. In the words of one participant, Gabriella, “*I think [the neuroscientific terms] justifies why you do things. And then you can explain it to people, yeah that's scientific. (Laughs) Like it's not just a crazy me thing, it's an actual thing that happens to most women who are pregnant*.” (Table 2: 1.1.2) Another participant, Alice, described her active search for emergent neuroscience research demonstrating links between pregnancy and cognitive deficit:

“*But this most recent pregnancy, I was struggling a lot with stress and brain fog… I was looking for academic research, “what are the effects of high levels of estrogen on cognition in women.”…. Looking for published research about what is there out there that might explain my subjective experience in terms of a scientific possible explanation...There's a bunch of stuff online that's kind of like, “mommy brain's not real.” It's real. It's absolutely real... This is an actual phenomenon. Not just women complaining. You know, not just women being lazy or whatever. But like an actual phenomenon that I can find no mention of in anything besides like pop reporting and that's why I started looking for, ‘is there any actual research out there about estrogen levels and cognition?’ That would legitimize what I'm subjectively feeling*.” (Table 2: 1.1.1)

The neurosciences are positioned to change our understanding of ourselves as “cerebral subjects” (Vidal, 2009). The explosion of brain research has solidified the brain as the organ that houses the “self.” For this participant, behavior is rooted in the brain and thus her understanding of herself is sought via neuroscientific proof. The brain rhetoric is validating: it relieves prior self-judgment and the presumed judgment of others who portend that she's “[just] complaining or “being lazy” (Table 2: 1.1.1). This language and base assumption is reflected in certain media portrayals that clarify mombrain is i.e., “backed up by science” (Gordon, 2020) and not just a “convenient excuse for forgetfulness” (Gordon, 2020). Alice's language suggests she has internalized the suspicion that women are unduly complaining or making convenient excuses for their incompetence. Becoming the “cerebral subject” (Vidal, 2009), however, is defense against this critique.

#### Mombrain as Stigmatizing: “[The] Longterm Performance Problem”

The other presiding reaction to “pregnancy brain” and “mombrain” was one of apprehension. These participants suggested that regardless of whether they had experienced memory challenges in the perinatal period—some had, others had not—they were uncomfortable with the framing of such experiences in neurobiological terms. To these women, compromised cognitive functioning was more aptly interpreted as ramifications of heightened multitasking or lack of sleep. For them, the popularization of brain rhetoric was a threat to the perception of their competence and to their wellbeing, personally, and interpersonally.

“*I feel like we hear a lot about [mombrain] in popular culture. I clicked on something on the Internet the other day, I think it was something on PET scans like, there's less activity in the hippocampus in women who've given birth for some number of years afterwards. I've heard of things like that. I know there might be some evidence to it… I hope it doesn't contribute to people's impression of me at work when I'm back after having a baby, et cetera. Yes, of course, like if I didn't sleep well, then I was tired the next day, but I took like exams and stuff, maybe like a year-and-a-half postpartum and I did just as well as I had done on previous ones, so I feel personally, I was fine. It's not the greatest concept if it's going to discourage people from either doing things at work or if it's going to affect other people's perceptions of them. Just pretending it doesn't [occur] seems okay for me. So that's what I'm going to do*.” (Table 2: 1.2.1)

Louise and others conveyed a conscious act of preferencing one explanation over another. This participant privileged a sleep narrative, choosing to ignore the brain narrative. This description reflects a dichotomization present in the public dialogue: the phenomenon in question—i.e., forgetfulness—is caused either by the brain or by chronic lack of sleep. This dichotomization may arise and be perpetuated at numerous points in the production and translation of a scientific finding. The design of the study itself may not take an integrative or “ecosocial” view of the brain (Kirmayer, 2019), but instead treat the brain in isolation from its environment, neglecting critical contextual factors that influence the results. In the translation and uptake of neuroscientific study, descriptive findings may be interpreted as causal. What is often absent from design or dialogue is the notion that “brains in question” as subjects of study do not exist in a vacuum, but in complex interaction with their surroundings. The narrative based in the brain and the narrative based in the social world are not at odds with each other, but are different levels and lenses on a particular phenomenon each with their own affordances and limitations.

Many of our participants were fearful of the stigma brain-based explanations could bear. Phoebe disclosed that she was “over-performing” at work during her pregnancy as a compensatory measure (Table 2: 1.2.6). She presumed that her colleagues would perceive her incompetent due to “pregnancy brain” and later, “mombrain.” This sentiment was echoed. Beatriz suggested that although she did not feel anyone “[held mombrain] against [her]” during her first pregnancy, she harbored feelings of personal inadequacy and was constantly in fearful anticipation that her brain would fail her: “*Oh my God, why did I forget, is because of my “mombrain”?”* (Table 2: 1.2.2). For these women, the anxiety of the brain-based explanation of the phenomenological experience revolved, in part, around the premise of seriousness and permanence.

“*When the term brain fog is used, it sounds like it's something that's less correctable... less competent versus if you say it's like hormonal changes or you're sleep deprived or it's the pregnancy: those are all things that come to an end fairly quickly. So they can't be used as a long term performance problem. Because they specifically write an article that talked about there being changes that lasted at least up to three years based on their follow-up period in the study*.” (**Table 2: 1.2.3**)

An explanation in terms of sustained alterations in neural architecture constructs what is felt as a prolonged and insurmountable obstacle as opposed to a passing physiological state. The attribution of the phenomenology to sleep deprivation has a clearer, more practically actionable solution than if the narrative focus is on changed brain morphology. For the highly cited paper, “Pregnancy leads to long-lasting changes in human brain structure” (2017) the researchers claim the observed structural alterations are connected to the “biological process of pregnancy rather than to experience-dependent changes associated with approaching parenthood” (Hoekzema et al., 2017). A methodological examination of the degree to which these researchers are able to solidly make this claim is beyond the scope of this paper. The public participation in neuroscience, however, tends toward non-critical acceptance and as the transmutations of research papers become more distal, it is possible that the likelihood for misconstrual of sound conclusions is heightened.

A few participants drew a connection between the rhetorical use of “pregnancy brain,” and “mombrain” to that of “Pre-menstrual syndrome (PMS).” Though they did not dispute the phenomenological experience of e.g., memory lapses, they were fearful that “mombrain” might be leveraged as a means to discredit via assumed inferiority to men.

“*But I, what I find frustrating [is that] there's this trope for so long about women can't be leaders because of our menstruation because when we have PMS, like we're crazy and wild. I think mom brain fits into the same thing where [the] narrative is compared against [a] male standard...And so the value and the power and the capacity for pregnant women to play this incredible role in society is downplayed. And instead, what, what gets projected out is, ah look she becomes a shitty employee... So I think it's just this patriarchal standard and it doesn't serve us*.” (Table 2: 1.2.4)

Another participant, Maya, expressed that she felt tension between denial and acknowledgment of the implications of “mombrain.” Maya's words highlight a common misconstrual. The studies purporting to show volumetric reductions in particular brain regions are not only contested but do not imply that cognitive deficits follow. Maya feels, however, the interpretation of biological difference gives way to a societal conception of female limitation and meaning-making through a strictly capitalist lens.

“*Around the brain fog first: the balancing of the narrative for me is the important thing. Cause it's like a big part for me. I don't like these hashtags, you know, hashtag brain fog, hashtag mom brain partially because of the impact that a lot of this stuff had on me in terms of my work, you know, and the unspoken sense of not being as competent…So I just feel this real tension between wanting to acknowledge that this is a very real thing, right. Where I'm just like, 'my memory was wasn't as good', you know, like there's many ways in which I wasn't as capable in terms of being productive in a sort of capitalist productive way. I was very more creative and more able to do certain things, but definitely less able to do others*.” (Table 2: 1.2.5)

This participant highlights the bind in which she finds herself, meriting a balancing act. To reject or downplay the feeling that her memory suffered during her pregnancy would be insincere, yet to acknowledge this phenomenon as #mombrain is to submit herself to a position of inadequacy by societal metrics.

### Theme 2: The Looping Effects of Biomedical Narratives

Epigenetic research establishes new meanings for perinatal mental health: the mental health of the mother impacts not only her, but her child. Research suggests that the experience of depression, stress, and anxiety during pregnancy may have negative effects on fetal growth and development **(**Arabin and Baschat, 2017; DeSocio, 2019), that maternal prenatal stress programs infant stress reactivity (Palma-Gudiel et al., 2015; Arabin and Baschat, 2017) and that high levels of circulating cortisol alter patterns of infant brain connectivity (Bock et al., 2014). Research points to the care a newborn receives bearing impact on the development of neural systems. The widely popularized pup-licking paradigm implicates maternal mental health and behavior toward the infant in the generation of differential responses to stress for that infant down the line (Meaney and Szyf, 2005). Though studies point to multifarious specific risks and affronts, actual impact to the child is defined by multifactorial and complex dynamics between both risk and protective factors. Attachment theories predate epigenetic findings, but the genetic lens—as opposed to the psychological one—may have a validating effect and increase the perceived seriousness and pressure felt by women who engage with this research. Women face a new moral imperative to monitor their perinatal mental health for the safety of the infant, constantly assessing the “normalcy” of their psychological state.

#### Ripples of Knowledge: “Concerned About My Mental Health”

As her group's discussion turned toward epigenetics, Louise reflected:

“*Your genes are supposed to be set in stone, except that there are environmental things that can cause changes in the gene that persist over the longterm. So like...if there's stress in pregnancy, like COVID, like with my daughter... like a big environmental event or multiple little ones, that can change your genes. Well, they might remain changed that way down the road for many years and maybe even passed on for the next generation. Which is I think where the interest in pregnancy comes from... pregnant women and stress and how it could negatively impact the baby... in regards to…some natural disaster that had occurred like either a flood or a fire somewhere*.” (Table 2: 2.1.1)

Participants had engaged with epigenetic research translations ranging from: the impact of food shortage on body mass of the descendants of individuals living in the Warsaw ghetto during the Second World War (Table 2: 2.1.2; 2.1.3), the repercussions of natural disasters like a massive ice storm that struck eastern Canada and New England in the late 1990s (Table 2: 2.1.3), the COVID-19 pandemic (Table 2: 2.1.1; 2.1.3; 3.2.7), intergenerational transmittance of trauma experiences (Table 2: 3.1.1; 3.1.4; 3.1.5; 3.1.6), and the impacts of compromised mental health issues (including stress, anxiety, and depression) (Table 2: 2.1.3–2.1.7) during pregnancy, specifically, which materialized as the most concerning theme for the majority of participants.

*I was actually worried about epigenetic effects in the baby. Worrying maybe that they would be more sensitive to stress or what have you. I wasn't worried about things like Down's Syndrome or developmental… I was confident in my physical health while I was pregnant. I was mostly concerned about my mental health and any potential epigenetic effects that would have on the baby…* (Table 2: 2.1.4)

Many of our participants had engaged with epigenetic research translations suggesting an association between perinatal mental health issues (e.g., stress, anxiety, and depression) and negative impacts for their children. This information was deeply unsettling. Analysis of womens' narratives reveal that, for a number of participants, engagement with epigenetic research translations precipitated a heightened level of awareness including increased self-monitoring and concern for mental and emotional life during the perinatal period.

*Well, I'm stressed out today because life is stressful. But I shouldn't be stressed cause that will hurt my baby. It ratchets up all of the stress that you're feeling.…there were some times that I was frightened and really angry and really unhappy and I was thinking I can't protect my baby from these feelings, from whatever's happening to me physiologically. So, I definitely did have those thoughts. What is the effect of this fight? This blowout? Me being frightened? Me being angry? Me being really hurt and I can't protect her from it*. (Table 2: 2.1.5)

Ordinarily, fluctuating emotional states may be dismissed as everyday ups and downs (Kirmayer and Sartorius, 2007). Pregnancy, as a period of constantly emergent change may present a wealth of these acute, transient moments of bodily distress. The recent widespread dissemination and uptake of epigenetic and neuroscientific research may offer a lens that constructs a situation where potentially transient bodily fluctuations and distress risk being experienced and reframed in more medicalized and “at risk” terms. When such acute yet fleeting experiences of stress occur during pregnancy, their ascribed meaning may now be influenced by the belief that such stress harms the child. Mechanistic descriptions of methyl groups and histone modifications authoritatively convey the effects of stress that transcend the maternal body as assaults to the infant. The stress has become more dangerous and sticky. The knowledge of the consequential severity of a stressed condition may increase a woman's bodily preoccupation, which may increase the salience and severity of the perception of stress, leading to further emotional arousal.

The narratives of our interlocutors expose this heightened level of awareness and self-monitoring induced by pre-emptive categories of “at-risk” that emerge as part of epigenetic research translation through cognitive-interpretative and social-interactional looping processes (Kirmayer and Gómez-Carrillo, 2019). Processes of biolooping at the intrasubjective level couple bodily enactment and physiology (Hacking, 2000; Kirmayer and Gómez-Carrillo, 2019) that can change the course of perinatal experience, leading to symptom amplification, heightened distress, and reduced functioning thereby reinforcing the very experiences that epigenetic research warns of. Through processes of classificatory looping at the intersubjective level the pre-emptive “at risk” becomes actualized through its mere potentiality as a category. Perinatal distress is not only exacerbated but the woman becomes one of a kind: an epigenetic risk factor for her offspring. In Hacking's conceptualization of classificatory looping, “kinds” of people emerge via the authority of expertise and classification systems of science (Hacking, 2000; Seligman, 2018). Hacking proposes that these two types of looping effects may occur simultaneously and be “mutually reinforcing” (Hacking, 2000, p. 109).

As epigenetic research findings leave the laboratory, enter the mainstream press, and manifest in various forms, nourished by numerous actors, their significance is reinforced, and they become ubiquitously established in the pop science realm. Once a woman becomes privy to this body of science and way of thinking, she is but a few clicks away from accessing a colossal number of its instances which can influence how she makes meaning of her experience, defines herself, and understands her relationship to her body, mental health, and child. Hacking's biolooping notion highlights the capacity for a “change in our ideas [to] change our physiological states” (Hacking, 2000, p. 109). Through the engagement with authoritative epigenetic narratives, prevalent across various media forms and medical locales, women's ideas and beliefs on this topic can come to shape their bodily sensations and states.

An enduring loop may not only increase self-monitoring but prompt the self-assessment or categorization as “sick”: a someone with a hazardous, pathological level of stress. Through this chain reaction (loop), a transient experience of stress may well reach a threshold and become disabling through a “vicious circle of symptom amplification and chronification” (Kirmayer and Sartorius, 2007). This bioloop is exposed by Gabriella's words:

“*I had so much trauma since January, my levels of cortisol were so elevated all the time and when I was working it was easier to be distracted by something so cortisol levels would come down but now my cortisol levels were so high all the time, all I could think about was, how is this going to affect her when she comes out? Right, because everybody tells you, you have to stay calm, you have to be so happy… I'm crying all the time, I'm loosing my mind, I don't know what's going on. And all I think about is, “cortisol is too high, I've gotta calm down*.” (Table 2: 2.1.6)

How does one find respite for this self-perpetuating loop of intensified self-monitoring and amplification of stress, worry, or pessimism? Charlotte, who had managed her anxiety with pharmacological intervention, discussed the dilemmas she had encountered in finding relief during her pregnancy. If she refrained from medicating and left her anxiety unchecked, the anxiety could harm her baby. Concurrently, she harbored concern about the potential impacts of medication: “*So it was kind of a battle to know this is probably not best for me to be on medication, but at the same time if I'm not then this is not going to be a healthy pregnancy for me*” (Table 2: 2.1.7). The experience of this participant reveals the double-bind consequences of epigenetic findings for mothers-to-be: which is more harmful? An “unhealthy” pregnancy and the epigenetic impact of manifested anxiety or the unknown ramifications for the child from medicating while pregnant?

#### Ripples of Risk and Diagnosis: “It's Going to Happen to Me”

Al-Gailani (2014) writes that the research interest, ease of uptake, and widespread establishment of folic acid as a necessary preventive measure for women of childbearing age was possible due to the construction of spinal bifida as an “urgent problem for the medical profession, charities, and society at large.” Like folic acid for its time, issues of mental health have captured popular attention in recent years, increasingly defined as public health emergencies. Not only is depression more widely viewed as a “free-standing, biologically-based” (Summerfield, 2006) brain disease, but, coupled with the lens of epigenetic and neuroscientific research related to “the maternal brain,” is also viewed as a disease that can have lasting biological impacts across generations. The affective heft of current discourse on the maternal brain may rely in part upon the context of society in the “grips of a mental health crisis” (The Centre for Addiction Mental Health, 2020).

The feeling that one's pregnancy or postpartum is abnormally unhappy or difficult can be reinforced by the increasing public awareness of depression as a grave, brain-based disease that afflicts many women. Postpartum depression, specifically, has great traction in public sphere. At the time of writing the following Instagram hashtags had a traction of #PPD (288k posts), #postpartumdepression (322k posts), #postnatalanxiety (22.8k posts), #normalizementalhealth (17.2k posts), #honestmomconfessions (122k posts). For someone experiencing some degree of postpartum distress, reading about the prevalence of depression and anxiety, engaging with research translations that confer a high truth status to the seriousness of mental disorders, or interacting with others' personal accounts of #PPD on social media, can either have a supportive, validating effect on their experience of distress as abnormally unhappy, or increase their attention to their distress and support self-diagnosis, or a mix of both.

It is possible that the siloes and echo chambers that the Internet, especially social media, fosters, lead to myopic engagements with a type of content and increase patterns of looping. Beatriz reflected on how her engagement with PPD narratives online had provoked considerable anxiety and contributed to the belief that she would develop PPD.

“*You do get flooded with all kinds of scary things. The talk about postpartum depression: it's so needed. It is. And of course, you know, you need to be aware of it, but just talking about having it was giving me so much anxiety that I was like every 15 days seeing a doctor to prevent postpartum depression that I never would have in the first place...So it does create needless anxiety. I was dealing with a lot of anxiety and I was hearing that having postpartum depression was gonna be a sure thing for me. My mom had it for me after birth. So I was like it's going to happen to me, I have it in my genetics. So I prepared. I was afraid of it. As a mom, everything you hear, you get so afraid. I would say that it's the news and everything that comes out of it. It's so sensationalist. As a mother hearing about epigenetics and all this sensation about it…*” (Table 2: 2.2.1)

Diagnostic labeling is a cultural artifact that can provide a meaning for hardship, an understanding of the seriousness of a condition, and a means of communicating its significance (Kirmayer and Sartorius, 2007). The act of taking on a diagnostic label can alleve distress associated with uncertainty and affords the individual a map of therapeutic possibilities and social consequences (Kirmayer and Sartorius, 2007). PPD was removed as a diagnostic category in the The Diagnostic and Statistical Manual of Mental Disorders, Fifth Edition (DSM-5), but the popular uptake and attention to postpartum depression as a unique and distinct affliction—served by campaigns to raise awareness, destigmatize its diagnosis, etc.—has meant that the PPD label still widely circulates idiomatically in society as part of a cultural vocabulary, despite its removal as a discrete psychiatric entity. The continued lay use of PPD to explain distress during the postpartum period may also be supported by the public understanding of depression—writ large—as a disease of the brain. Neuroscientific inquiry on the perinatal period and the popularization of the “pregnant brain” or “mombrain” as a particular “kind” of brain, may contribute to the ongoing PPD rhetoric in society. To what degree do the descriptions of neural remodeling during pregnancy and interpretations about their meaning (that disseminate across the Internet) support the idea that PPD is an *expected* byproduct of such structural and functional brain changes brought about by pregnancy? The conclusion of one scientific article explicates an alleged connection between documented pregnancy and postpartum brain plasticity and a predisposition to mental disorders:

“*A compelling body of evidence in healthy women and other female mammals confirms that, during pregnancy and the postpartum period, hormones and sensory interactions with the offspring relate to complex structural and functional changes in the brain….Although this maternal brain plasticity facilitates a higher purpose—the continuation of the species—it is not necessarily innocuous and predisposes the mother or mother-to-be to peripartum mental disorders.” (Barba-Müller et al.*, *2019**)*

Seeking out readily available biomedical translations that discuss prevalence of PPD^2^ or point to connections between documented changes in the “maternal brain” and compromised mental health, as well as interaction with others' PPD narratives, may all be factors that increase preoccupation and self-monitoring of affective states and bodily sensations that are then identified, labeled and given meaning in psychiatric terms. The comparison, internalization and interaction with boundless expressions, descriptions, and communications of distress online may serve as social reinforcement that catalyses the symptom amplification characteristic of biolooping and assumption of a sick role, characteristic of categorical looping.

Our data speaks to the possibility that the web of epigenetic and neuroscientific translations and the sociocultural environment of the digital sphere—an increasingly dominant space—may be exacerbating women's experience of emotional distress or the propensity and ease at which individuals may fall into looping trajectories.

### Theme 3: Imprints of Past Experience and the Management of the Future

#### Translational Trauma

The allure of epigenetic narratives may rest on the following notion: we may not have control over our genes, but we do have control over the experiences that influence expression of our genes. But we cannot control the past experiences of our parents or grandparents. So, what then? Though at its essence, epigenetic research points to biological flexibility, a prominent rhetoric often propagated in the public sphere is one of fixity, not so dissimilar to the deterministic narrative of genetics. Preliminary epigenetic research exploring biological transference of trauma, specifically, is a subject that has received considerable media attention. Science Magazine, published a piece titled, “Parents' emotional trauma may change their children's biology. Studies in mice show how.”

“*But today the hypothesis that an individual's experience might alter the cells and behavior of their children and grandchildren has become widely accepted...“This is really scary stuff. If what your grandmother and grandfather were exposed to is going to change your disease risk, the things we're doing today that we thought were erased are affecting our great-great-grandchildren*”” (Curry, 2019)

The evidence of intergenerational transmittance at the layer of the epigenome was a subject of concern for a handful of participants whose family history was mired in hardship. Maya shared:

“*Um, similarly I heard something again, I don't know how verified it is. Someone sent me an article [about epigenetics]. I think it is that their experiences or traumas, this got imprinted on their DNA in some way. And that that gets passed down. And I remember being, first of all, it just seems so sci-fi that, really, it like sticks to your DNA, that experience? Then I got nervous cause I was like, Oh my God. Thinking about my grandmother's experiences. And then thinking about my own son and, and you know, my partner's mother and then my mother and just being like, I have no control over this, you know, they've been through so much, he's going to experience that on some level maybe*.” (Table 2: 3.1.1)

In our sample, it appeared that women who had engaged with translations of epigenetic research discussing the biological inheritance of trauma felt demoralized by this knowledge. The perceived inability to control or act upon past experience with the subsequent feeling of becoming a powerless vector of troubled histories was a source of distress. While certain participants felt distressed by what was understood to be permanent, inactionable harm caused at the level of the epigenome, others invoked a contrasting narrative of flexibility and plasticity. Victoria promoted a narrative of rectification, advocating for the individual's agency to write past wrongs and the potential to optimize action to effect positive change. The malleability of the “plastic brain” figured in this narrative, as proof of the possibility for remediation and opportunity.

“…*The brain is plastic. You can always change it, you know, in a positive or wrong way, but it can be changed...There's also a lot of negativity about epigenetics. We forget...we can also do positive things. Life gives us the chance to change it again and to make it right*.” (Table 2: 3.1.2)

The notion of the plastic brain was used by this participant as a means to console or relieve other women's distress over the epigenetic inheritance of trauma, there was a concurrent notion that specific windows—“the early years”—of brain development are very important, demanding meticulous action for goals of reparation or enhancement. The correction of issues in the past is conditional upon one's actions as a mother.

“*I read a lot and most of it has to do with neuroscience and the way the brain is shaped and how the early years are super important… Even though you don't have your dream birth or the best, pregnancy, the thing is that you can change it, you can…you have every day to make it better and every day to achieve a positive experience with your kids*.” (Table 2: 3.1.3)

Beatriz, with a degree in biology, was conscious of the tensions and binds of rhetorical themes that emerge across epigenetic translations of science. She articulated her understanding of the multifactorial nature of epigenetic impact: the complex interaction of risk and protective factors. Beatriz shared that even though her background and training afforded what she believed was a sophisticated ability to unpack and critically analyse scientific findings, she nonetheless found herself affected by headlines and various translations of biomedical research, her scientific acuity fading out of focus as she became absorbed with the popular medical discourse as a mother-to-be.

“*They take a scientific paper, they take one piece of information, they make it a big headline. And then they talk about it like it was the end of your life. Your child is going to be an abuser or is going to be a rapist because your grandfather was. It's like, it's they take it out of context and it creates so much anxiety. And it's like, no, you know, it's such a small thing. The body has so many protection mechanisms. That it's not because something happened in the past, they're doomed to happen again. So balancing that perspective with being in the middle of the feeling and receiving all that information, you know, it's kind of hard for me and I kind of forgot about my theory, forgot about what I knew. I forgot about the deeper analyses and inside me I was like, Oh my God. And I had to remind myself, no, I dyed my hair, but my baby is going to be fine. And my grandmother killed herself when she was 40, but I'm going to be fine. My baby's going to be fine. It's a lot of work. I find that it's a lot. It's intense*.” (Table 2: 3.1.4)

The translation environment of click-bait headlines, sensationalized scientific findings, and the dichotomy of simultaneous fatalistic and responsibilizing language was a source of anxiety, and she has to do the “work” to make sense of it and act accordingly.

#### Responsibilization of the Mother-To-Be

In their examination of the political and practical implications of epigenetic science, Wastell and White (2017) evoke Schrödinger to illustrate the tensions the epigenetic narrative poses:

“In freeing us from determinism, this form of genetics creates a space for benignant social engineering. Schrödinger refers to its possibilities as ‘beautiful, elating, encouraging and invigorating’ (p107), but these enticing prospects may also create minatory moral hazards.” (Wastell and White, 2017, p. 20)

Wastell and White (2017, p. 20) argue that “good enough parenting” (19) is no longer good enough in a context where a mother's behaviors, actions, and emotions are “etched indelibly on the infant's brain and written into the molecular activities of its cells” (19). The epigenetic narrative places the responsibility on the mother to prevent damage to her infant via (a false notion of) control of micro and macro aspects of herself and her environment, and thus the mother becomes both an object of her own self-monitoring and an object to be controlled socially and biomedically. She holds the responsibility to protect her child from trauma or other nefarious influences such as her own behavior, her diet, and her mental health. There are numerous instances of this “with great power comes great responsibility” perspective circulating in the popular sphere. “*You can positively influence your epigenome,”* a slide in a TEDx video “Epigenetics and the influence of our genes | Courtney Griffins|TEDxOU” that has been viewed over half a million times (TEDx Talks, 2012) reflects this perspective: it is within a woman's power to do right (or wrong) and thus she is measured in the efficacy in which she promotes beneficial outcomes for her child. The manifestation of this denouement affords a context of monitoring by self or state.

“*It made me angry at our society. This is ridiculous. It's like we have information telling us that having elevated cortisol levels and super high stress is absolutely associated with negative outcomes. But, there's no support for you...But, keep going and eat a fucking salad*.” (Table 2: 3.2.1)

Women find themselves in numerous binds vis à vis their biomedical information consumption during the perinatal period. Our interlocuteurs reported the desire to self-educate to be informed and equipped with expert knowledge. Though participants sought the outputs of emergent biomedical and scientific research, they struggled with the navigation of its translations—itself a unsettling affair—and found their interaction cognitively and affectively straining. In response to these often lose-lose engagements with biomedical and cultural constructions of the perinatal period, some women found themselves stressed, others all together disengaged, but others acknowledged interpreting the narratives communicated to them in a flexible manner: “*I use it to my benefit when it works out, like a horoscope. When it doesn't work out, I don't like it*” (Table 2: 1.1.3)

Overall, participants felt that the outputs of current scientific inquiry into female reproduction—particularly from neuroscience or epigenetics—placed enormous pressure on them as individuals to affect change or control variables in their lives with oftentimes limited societal support. One participant, Teresa, actively refrained from engaging with the Internet during her pregnancy upon the realization that the pressure of responsibilizing messaging across biomedical research translations was creating distress for her.

“*I think that there was some part of me that was very stubborn about resisting that kind of information because I felt like that it wasn't something that I should have to take on: that I should have to be worrying about every single thing I thought or felt or did. And so there was some part of me that was very rebellious that way. And then every once and a while I would get sucked in and it would cause me this terrible anxiety and I would have to go back and sit and think about what do I want, how do I feel?*” (Table 2: 3.2.2)

Teresa describes herself as being “rebellious” for avoiding engagement with biomedical research translations online. This notion of “rebellion” implies an authority to which she is expected to obey or expectations of norms or rules that she rejects. The preeminence of medicalized discourse around pregnancy and the availability and accessibility of medical and scientific expert knowledge has been shown to beget an internalized responsibility to self-educate (Marshall and Woollett, 2016; Tiidenberg and Baym, 2017). Teresa seems to be rebelling against the reach of authoritative science into her pregnancy experience. She seems to be resisting the expectation that it is her duty, responsibility to follow emerging research findings and current evidence-based recommendations. Women experience individual responsibilization to be informed and to act upon said information, whether it regard the mitigation of self- or externally-imposed expectations of mombrain-related incompetence, the necessitation of risk management and prevention of epigenetic insult through self-monitoring, the management of mental health, or micro scrutiny of behavior, actions, emotions, exposures, consumptions, etc. The web of various actors, vectors, and recipients of biomedical and pop culture pregnancy discourse has assisted in the creation of a climate where women are monitored by self and other.

“*The other frustration for me which is less personal, it's more social, was this information should be used to make structural changes to lessen stressors on people's lives…We seem to have this idea that regardless of the science whether it's positive things you can do or negative things you shouldn't do, it still places enormous expectation on individuals*.” (Table 2: 3.2.3)

## Discussion

Focused discussion revealed that many women find themselves trapped in a double bind with conflicting messaging and situated in various no-win situations when attempting to inform their choices as mothers and make sense of their perinatal experience.

A double bind (Bateson, 1972) is a situation of conflicting narratives or demands that the individual is unable to resolve or opt out of. The uptake of translations of neuroscientific findings on structural brain changes during the perinatal period has created such a bind for mothers: By accepting “pregnancy brain” as real, women compromise the perception of their competence. By dismissing pregnancy brain as not real, emotional, and cognitive challenges remain illegitimate, while women are faced with a social reality characterized by numerous demands, expectations, limited societal support, and inevitable exposure to social judgment as a pregnant woman and mother.

The experiences, emotions, and perspectives of our participants are reflective of the value and import of examining the dynamic life of a scientific discovery as it leaves the laboratory and is translated on entry to public spheres. Interconnected channels and feedback loops of the laboratory, science journalism, public opinion and reception, public and private funding bodies, influence broader “citation practices,” and paths of research. With social networking and a plethora of new media platforms, citations, or translations of research emerge in many forms across a diversity of channels.

Overall the media environment in which these women encounter biomedical perspectives and prescriptions around the perinatal period is a quagmire. Participants expressed a thirst for information during their pregnancies and into early stages of motherhood: having the information provides a sense of control and agency but oftentimes the information is equivocal and difficult to make sense of. Women encounter warnings of looming dangers to their children largely beyond their control while placing the onus on them as individuals without much scope of societal support.

Translation of epigenetic science thus introduces another bind. Offering leverage on the sticky predicaments and histories of your ancestors, it inflates the weight of this inheritance and puts one to work to undo what has been done without guarantee. Cognizant of this power to harm and to protect, the value of plasticity and choice afforded by this body of knowledge risks being lost to self-monitoring, responsibility and stress about stress.

Capturing a social anxiety around the impacts of the pandemic on infants and children, in May of 2020, the Canadian broadsheet newspaper, *The Globe and Mail* published an article entitled “Will pandemic babies live with the effects of their mothers' stress?” (Ungar and King, 2020). It is likely that the intensity, duration, and global scale of this event may heighten the attention of pregnant women to prenatal maternal stressors understood to compromise the developmental trajectories of their children via epigenetic and neurobiological pathways.

Future research should explore how the context of the COVID-19 pandemic is impacting on the actual experiences of women during the perinatal period, but also on the ways in which these experiences are being framed in terms of existing public health messaging drawn from biomedical research on the imprint of the environment on genes and the brain. The women whose narratives are the foundation of this paper shared their experiences and reflections across three focus groups held in late summer and fall of 2020. Months had elapsed since the COVID-19 pandemic first became front-and-center in life in North America. The wider realities of this context impacted the pragmatics such as recruitment process and focus group method, but also, and potentially the findings of this study. Earlier high-profile research initiatives such as the widely publicized “Project Ice Storm^3^” have reported that *in utero* exposure to prenatal maternal stress from an isolated independent stressor—in this case, the 1998 Quebec Ice Storm—resulted in significant long-term effects on “temperament, parent- and teacher-rated behavior problems, motor development, physical development, and IQ, attention, and language development,” (Projet Verglas) the majority of which the research team purports persist past 19 years-of-age. The events of the 1998 Ice Storm left individuals without electricity for up to 45 days; at the time of writing, the COVID-19 pandemic has had profound impacts on numerous domains of life in North America for a year's time. How might women in diverse contexts be making sense of the length and gravity of this “event”?

In conjunction, new mothers may be concerned about the future behavioral development, such as compromised sociality, of their babies. Future research is needed to examine women's uptake, attitudes and feelings toward this specific area of COVID-19 related research, and the ways in which these interpretations are framed in terms of biomedical knowledge.

The women in our study were engaging with knowledge translations of the authoritative scientific bodies of epigenetics and neuroscience and applying these “imaginaries” (Meloni and Testa, 2014) to their own trajectories, experiences, and life predicaments. These translations are not innocuous. If a woman's expectations include that she will manifest inevitable mombrain-related incompetence or the prior that her level of stress puts her at high risk of harming her child's development, or the presupposition that she will develop postpartum depression from pregnancy-related changes in her brain—to what degree does the shaping of mindset and expectation by these presiding biomedical and cultural rhetorics engender the maladaptive changes in subjective experience, behavior, and physiology that are so feared?

The hope of objectifying certain phenomenological experiences and states biological proof continues to reignite rather than rid the tropes of earlier bodies of knowledge that stigmatized and responsibilized women, mothers, and the female body as such has clearly failed. Instead of liberating mothers, patients and others from this sense of moral or behavioral failures by providing corporeal difference and material validation, the notion that the brain is aberrant and the moral imperative to act on the body, though framed as agency for some, clearly replicates aspects of this historical stigmatization and responsibilization. Such responsibilizing narratives resonate with the notion of “mommy economicus” (Thornton, 2014), “a new mutation of the socially prescribed ‘good mother’” offered up by “mombrain” brain discourses that stem from research on neuroplasticity. The maternal brain as a “kind” of brain has not only conjured maternal brain-related vulnerabilities or deficits such as “mombrain”-related-amnesia, but has also engendered dichotomous messaging speaking to maternal brain-based superpowers afforded by the unique window of maternal neuroplasticity (Thornton, 2014). “Mommy economicus” casts further light on this tension between the dichotomized rhetorics of both neuroplasticity and epigenetics: a sense of fixedness or determinism—not so different than implications of genetics—or a privileging of personal empowerment, individual choice, and self-fashioning characteristic of neoliberalism and postfeminism (Gill, 2007; Vavrus, 2007; Ehrenberg, 2011; Gill and Scharff, 2013; Thornton, 2014).

Our participants' engagement with brain science was positioned between a search for determinism to legitimize their challenges and the moral burden of choice. Their accounts demonstrate how neurobiological and epigenetic knowledge contribute to a particular “regime of truth,” one in which—through molecularization of pregnancy and child development—a typical passage of life becomes saturated with “susceptibility,” “risk,” and the imperative to preemptively make “healthy” choices, in turn redefining and shaping the experience of what it is to be a “good,” “healthy,” or “responsible” mother/to-be. The illusion of agency conferred by shaping brains or imprinting DNA is continually shadowed by a sense of failure, disappointment, and vicious cycles of anxiety.

## Data Availability Statement

The raw data supporting the conclusions of this article will be made available by the authors, without undue reservation.

## Ethics Statement

The studies involving human participants were reviewed and approved by The Institutional Review Board of McGill University. The patients/participants provided their written informed consent to participate in this study.

## Author Contributions

ION-S aided in conceptualization of the study, lead data collection and analysis, drafted the manuscript, contributed to conceptual work and editing of manuscript. AG-C was involved in intellectual conception, helped with interpretation of data, contributed to drafting, revising, and editing the manuscript critically. SC conceived of the study design, aided data collection, and made contributions to conceptual work and editing of the manuscript. All authors contributed to the article and approved the submitted version.

## Conflict of Interest

The authors declare that the research was conducted in the absence of any commercial or financial relationships that could be construed as a potential conflict of interest.

## Footnotes

^1^Parenting, Pregnancy, and Epigenetics. *What is Epigenetics?* Available online at: http://www.whatisepigenetics.com/topic/parenting-pregnancy-epigenetics/ (accessed December 11, 2020).

^2^Barba-Müller et al. (2019) state “The most common [mental problem] is postpartum depression (PPD), with an estimated 11–20% of new mothers suffering from minor and ~7–14% from major depression (Gavin et al., 2005; Almond, 2009; Kirmayer and Gómez-Carrillo, 2019).”

^3^*Project Ice Storm Projet Verglas*. Available online at: https://www.mcgill.ca/projetverglas/icestorm (accessed February 25, 2021).
